# Erfahrungen von Menschen mit Fluchtgeschichte bei der Inanspruchnahme der Gesundheitsversorgung in Deutschland – Erkenntnisse einer qualitativen Studie

**DOI:** 10.1007/s00103-022-03614-y

**Published:** 2022-11-29

**Authors:** Anna Christina Nowak, Claudia Hornberg

**Affiliations:** 1 Fakultät für Gesundheitswissenschaften, Universität Bielefeld, Universitätsstr. 25, 33615 Bielefeld, Deutschland; 2 Medizinische Fakultät OWL, Sustainable Environmental Health Sciences, Universität Bielefeld, Bielefeld, Deutschland

**Keywords:** Flüchtlinge und Asylsuchende, Gesundheitssystem, Zugangsbarrieren, Handlungsstrategien, Qualitative Methoden, Refugees and asylum seekers, Health System, Access barriers and facilitators, Action strategies, Qualitative methods

## Abstract

**Hintergrund:**

Menschen mit Fluchtgeschichte sind mit einer Vielzahl von rechtlichen, strukturellen, administrativen, kulturellen und sprachlichen Barrieren im Zugang zur Gesundheitsversorgung konfrontiert. Derzeit liegen jedoch wenig Daten zu gesundheitlichen Bedarfen und Bedürfnissen von Geflüchteten vor. Insbesondere ihre subjektiven Erfahrungen im Zugang zur Gesundheitsversorgung sind bisher wenig berücksichtigt worden.

**Ziel der Arbeit:**

In diesem Beitrag sollen die subjektiven Erfahrungen von Menschen mit Fluchtgeschichte beim Zugang zur Gesundheitsversorgung und bei deren Nutzung beschrieben werden. Strategien bei der Bewältigung von Herausforderungen werden dargestellt.

**Methoden:**

Die Rekrutierung der InterviewpartnerInnen erfolgte anhand von quantitativen Querschnittsdaten, die im Rahmen einer Studie zur Gesundheit von Geflüchteten (FlüGe-Gesundheitsstudie) erhoben wurden. Personen, die einer erneuten Kontaktaufnahme zugestimmt hatten, wurden telefonisch kontaktiert. Es wurde eine im Hinblick auf Alter, Geschlecht, Nationalität, Gesundheitszustand und Inanspruchnahmeverhalten heterogene Teilstichprobe (*n* = 18) mittels dolmetschergestützter problemzentrierter Interviews nachbefragt. Die Datenauswertung erfolgte in einem deduktiv-induktiven Prozess.

**Ergebnisse und Diskussion:**

Die wenig migrationssensible Gesundheitsversorgung von Menschen mit Fluchtgeschichte ist geprägt durch Sprachbarrieren, Orientierungslosigkeit, Ablehnungserfahrungen, Fehlbehandlungen und strukturelle Barrieren. Fremdheitserfahrungen in und mit dem Gesundheitssystem können dazu führen, dass die Versorgung nicht (mehr) oder nur verzögert in Anspruch genommen wird. Gleichzeitig werden individuelle Strategien von Geflüchteten und VersorgerInnen angewendet, um den genannten Herausforderungen zu begegnen.

## Abstract (en)

**Background:**

Refugees and asylum seekers face a variety of legal, structural, administrative, cultural and linguistic barriers in accessing healthcare. However, there is currently a lack of available data on the health needs of refugees and asylum seekers. In particular, their subjective experience in accessing healthcare has not been considered sufficiently.

**Aim of the study:**

This paper explores the subjective experiences of refugees and asylum seekers in accessing and using healthcare. Strategies for dealing with potential challenges are presented.

**Methods:**

The recruitment of interview partners was based on a quantitative cross-sectional study collected as part of a study on the health of refugees (FlüGe health study). Persons who agreed to be contacted again were contacted by telephone. A heterogeneous sub-sample (*n* = 18) with regard to age, gender, nationality, health status and utilisation behaviour was interviewed using interpreter-supported problem-centred interviews. Data were analysed using a deductive–inductive approach.

**Results and discussion:**

The migration-insensitive healthcare for refugees and asylum seekers is characterised by language barriers, disorientation, experiences of rejection, mistreatment and structural barriers. Experiences of foreignness in the healthcare system can lead to delayed care seeking or underusage of healthcare systems. At the same time, individual strategies by refugees, asylum seekers and healthcare providers are used in order to meet these challenges.

## Einleitung

In den Jahren 2015 und 2016 wurden in Deutschland steigende AsylbewerberInnen-Zahlen registriert [1], die das Sozial- und Gesundheitssystem vor große Herausforderungen gestellt haben. Es ist bekannt, dass geflüchtete Personen vulnerabler gegenüber Infektionskrankheiten, chronischen und psychischen Erkrankungen sind [2–4]. Eine damit verbundene Gesundheitsdeterminante ist der Zugang zur Gesundheitsversorgung [5]. Trotz der erhöhten Vulnerabilität gegenüber unterschiedlichen Erkrankungen liegen formal-restriktive, administrative, informationelle und sprachliche Barrieren im Zugang zur Gesundheitsversorgung vor [6, 7]. Im europaweiten Vergleich schneidet Deutschland dabei auf rechtlicher Ebene eher restriktiv ab [8]. So wird der Zugang zur Gesundheitsversorgung für Asylsuchende in den 18 Monaten des Asylverfahrens sowie für Menschen ohne Papiere über das Asylbewerberleistungsgesetz (AsylbLG) auf die Behandlung von akuten Erkrankungen und Schmerzzuständen begrenzt. Die Behandlung chronischer und psychischer Erkrankungen ist vielfach eine Ermessensentscheidung der Sozialbehörden [9]. Dies ist sowohl aus einer (menschen-)rechtlichen als auch einer ethischen Perspektive kritisch zu beurteilen [10, 11].

Auch nach der Zuerkennung eines Schutzstatus oder nach Ablauf der 18 Monate bleiben administrative, sprachliche, kulturelle und strukturelle Barrieren bestehen [7, 12–15]. So erleben Geflüchtete Ungleichheiten im Versorgungszugang [12, 15] und berichten häufiger von ungedeckten medizinischen Bedarfen [16]. Dabei zeigen sich sowohl Herausforderungen beim Erstkontakt mit dem Versorgungssystem als auch in der Weiterversorgung durch Sprach- und Informationsbarrieren [17], Diskriminierungserfahrungen [17, 18], fehlende soziale Unterstützung [19] und negative Erfahrungen mit der Gesundheitsversorgung [17, 18]. Insbesondere die Kommunikation zwischen Professionellen des Gesundheitswesens und Geflüchteten wird in der Literatur als Herausforderung im Zugang zum Versorgungssystem benannt [20]. Für eine umfassende Gesundheitsversorgung sind deshalb die verbale und nonverbale Kommunikation, der Einsatz von DolmetscherInnen sowie kulturelles Verstehen und Verständnis Schlüsselelemente [20]. Unter anderem durch rechtliche Restriktionen und fehlende Finanzierung von DolmetscherInnen im System der Gesetzlichen Krankenversicherung [21] werden Menschen mit Fluchtgeschichte damit zu „Nicht-Bürgern“ [22, S. 452], weil ihnen ein gleichberechtigter Zugang zum Versorgungssystem verwehrt ist. Durch eine Vielzahl von Fremdheitserfahrungen im Zugang zur Gesundheitsversorgung und bei deren Nutzung können zudem psychosoziale Krisen entstehen [23], die aufgrund der vorliegenden Zugangsbarrieren durch die Gesundheitsversorgung nicht aufgefangen werden können.

In Deutschland gab es in den letzten Jahren deutliche Bestrebungen, die Datenlage zur Gesundheit und Versorgung von Geflüchteten zu verbessern (z. B. [24]). Es liegen aber weiterhin wenig repräsentative Daten zu gesundheitlichen Bedarfen und Bedürfnissen vor [25, 26]. Insbesondere aus der Perspektive der Geflüchteten selbst [27] besteht hier großer Forschungsbedarf. Ob die Gesundheitsversorgung als bedürfnisgerecht erlebt wird, lässt sich bisher nicht adäquat beantworten [28]. Deshalb sollen im Rahmen des Beitrags die Erfahrungen von Menschen mit Fluchtgeschichte mit der Gesundheitsversorgung nachgezeichnet und die Handlungsstrategien zum Umgang mit diesen Herausforderungen dargestellt werden.

## Methoden

Die vorgestellten qualitativen Ergebnisse basieren auf den Daten der Dissertation der Erstautorin, die im Rahmen des NRW-Forschungskollegs „FlüGe – Herausforderungen und Chancen globaler Flüchtlingsmigration für die Gesundheitsversorgung in Deutschland“ an der Universität Bielefeld erhoben wurden (vgl. [29]). Zu Projektbeginn wurde – nach positivem Ethikvotum durch die Ethikkommission der Universität Bielefeld – u. a. eine Querschnittsstudie zur Gesundheit von Geflüchteten (FlüGe-Gesundheitsstudie) durchgeführt (vgl. [30]). Anhand der quantitativen Daten erfolgte anschließend die Rekrutierung potenzieller InterviewpartnerInnen aus Gemeinschaftsunterkünften und (betreuten) Wohnunterkünften in einer Region in NRW. Um die Varianz und Heterogenität bei der Inanspruchnahme der Gesundheitsversorgung von Menschen mit Fluchtgeschichte abbilden zu können, wurden im Sinne eines qualitativen Stichprobenplans [31] folgende Personen befragt:

- Frauen und Männer unterschiedlicher Altersgruppen, die Arabisch, Farsi und Kurdisch-Kurmandschi sprechen; die Sprachgruppen wurden ausgewählt, weil ein Großteil der 2015 und 2016 nach Deutschland Geflüchteten aus Syrien, Afghanistan und dem Irak kommen;
- Personen mit einem guten, mittleren und schlechten Gesundheitszustand;
- Personen mit unterschiedlichem Inanspruchnahmeverhalten (hoch, mittel, gering) des Versorgungssystems, um unterschiedliche subjektive Erfahrungen mit der Gesundheitsversorgung abzubilden;
- Personen, die die Versorgung bisher noch nicht aufgesucht haben, wurden nur befragt, sofern sie angaben, dass sie Probleme beim Zugang zum Versorgungssystem hatten, oder über einen schlechten bzw. sehr schlechten Gesundheitszustand berichteten, da dann davon ausgegangen werden konnte, dass die Versorgung nicht bedürfnisgerecht war.

Es wurden 50 potenzielle InterviewpartnerInnen in den quantitativen Daten identifiziert. Davon konnten 32 Personen kontaktiert werden. Von 18 Personen lagen unvollständige Kontaktdaten bzw. keine Einwilligung zur erneuten Kontaktaufnahme vor. Insgesamt konnten 7 Frauen und 11 Männer für ein Interview rekrutiert werden (Rücklauf: 56 %).

Die Interviews wurden im Zeitraum August bis Dezember 2018 durchgeführt. Die Befragung der InterviewpartnerInnen erfolgte dolmetschergestützt mittels „problemzentrierter Interviews“ [32]. Dafür wurde ein Leitfaden entwickelt, der unter anderem Fragen zu gesundheitlichen Problemen, dem aktuellen Gesundheitszustand und möglichen Einflussfaktoren auf Gesundheit und Krankheit, den Erfahrungen mit der medizinischen Versorgung sowie möglichen Herausforderungen und damit verbundenen unerfüllten gesundheitlichen Bedürfnissen enthielt. Der Interviewleitfaden wurde an die Gesprächssituation angepasst, um eine Offenheit gegenüber den Themen der Befragten herzustellen.

Zur Durchführung der Interviews wurden SprachmittlerInnen ausgewählt, die über muttersprachliches Niveau in Arabisch, Farsi und Kurdisch-Kurmandschi und über Dolmetschererfahrung verfügten. Sie wurden in einer 2‑tägigen Fortbildung zu Gesprächstechniken, Besonderheiten des trialogischen Gesprächs, Dolmetschen im qualitativen Interview, dem Leitfaden sowie Krisenmanagement geschult. Während der Interviews wurde das sogenannte Gesprächsdolmetschen [33] angewendet, bei dem portionierte Gesprächspassagen abschnittsweise und zeitversetzt in die andere Sprache übertragen werden. Um eine möglichst natürliche Gesprächssituation herzustellen, sollten die SprachmittlerInnen lediglich Sinnzusammenhänge übersetzen und Erzählungen möglichst nicht unterbrechen. Die Interviews wurden nach schriftlicher und mündlicher Zustimmung digital aufgezeichnet. Die Transkription der Interviews erfolgte in 2 Schritten. Zunächst wurden sowohl die fremdsprachigen als auch die deutschsprachigen Anteile wortwörtlich transkribiert. Anschließend wurden die fremdsprachigen Interviewanteile in die deutsche Sprache übersetzt, um den Interviewverlauf und den Übersetzungsprozess in Nachgang reflektieren zu können [34]. Die Auswertung der Interviews erfolgte mit der inhaltlich strukturierenden qualitativen Inhaltsanalyse nach Kuckartz [35] unter Zuhilfenahme der Datenanalysesoftware MAXQDA Version 20 (VERBI GmbH, Berlin, Deutschland). Einen Überblick über das erstellte Kategoriensystem gibt Tab. 1.

**Table 1:** 

Hauptkategorie	Subkategorie	Beispielzitat
Fremdheit erleben	Sprachbarrieren	„Wenn ich zu einem deutschen Arzt gehen muss, verstehe ich ihn nicht. Bis ich ein Wort verstehe, verpasse ich zwei Wörter. Falls es kein Dolmetscher gäbe, wäre es sehr schwierig“ (I4: 546)
	Orientierungslosigkeit	„Wir wissen nicht wie die Deutschen, wo es einen fähigen Arzt gibt, oder sage ich mal, einen guten Arzt“ (I5: 2)
	Grenzerfahrungen	„Es ist sehr schwer, wenn man die Sprache nicht sprechen kann. Man kann auch nicht immer die Menschen an sich ketten, um für sich zu dolmetschen. Ich wusste nicht, wann immer mein Arzt da war. Als er gekommen ist und versucht hat mir zu erklären, konnte ich nicht verstehen und er ist auch deswegen immer weggegangen“ (I2: 758)
	Versorgungs(um)brüche	„… aber für mich hat sich [die Akutpsychiatrie] wie ein Gefängnis gefühlt, deshalb bin ich nach einigen Tagen aufgebrochen. Ich wollte nicht mehr dableiben, weil es alles nur noch schlimmer gemacht hat“ (I6: 226)
Fremdheit bewältigen	Strategien der Interviewten	„Ich sage ein Wort, meine Tochter sagt ein Wort, meine andere Tochter sagt ein Wort. Wir verständigen uns“ (I4: 357)
	Strategien der VersorgerInnen	„Wir haben uns bei Ärzten nie fremd gefühlt. Wir beherrschen die Sprache zwar nicht, aber wir sind gut aufgenommen worden. Die Behandlung und der Umgang waren sehr menschlich. Es lief natürlich alles über einen Dolmetscher. Es war insgesamt sehr gut“ (I2: 983)
	Unterstützung durch das Versorgungssystem	„An dem ersten Tag, wo ich operiert wurde, blieb der Dolmetscher [vom Sozialamt] drei Stunden mit mir. Er hat gesessen und auf mich, bis die OP fertig war, gewartet. Und hat mir alles gründlich erklärt. Er hat mir sogar die Salbe von Apotheke gekauft. Er war mir sehr gut“ (I2: 305)

## Ergebnisse

### Herausforderungen im fremden Gesundheitssystem

In den Interviews wurde deutlich, dass Sprachbarrieren die Bedürfnisgerechtigkeit mindern und Orientierungslosigkeit, Grenzerfahrungen sowie Versorgungs(um)brüche bedingen. Selbst- und Mitbestimmung wurden als eingeschränkt empfunden, weil Anliegen und Bedürfnisse nicht geäußert werden konnten (I6: 238; I15: 91). Es wurde von Missverständnissen gesprochen, die nicht behoben werden konnten, weil keine gemeinsame Sprache gefunden wurde und keine alternativen Strategien eingesetzt wurden:

„Ich habe kaum mit ihnen gesprochen, am Anfang habe ich versucht zu schildern, dass ich Alkoholprobleme und toxische Gedanken habe [Dolmetscher: Auf Deutsch?]. Ja. Und die meinten, dass die mir Medikamente wie Pillen geben werden“ (I6: 238).

Der Versuch, „zu schildern“, scheiterte hier. Die Sprachbarriere führte dazu, dass der Interviewpartner seine Bedürfnisse in der psychiatrischen Versorgung nicht adäquat mitteilen konnte. Zudem erfuhr er keine empathische Zuwendung seitens der VersorgerInnen, was schließlich zu einem Versorgungsabbruch führte.

2 Interviewte sagten, dass Ihre Behandlung abgelehnt wurde, weil eine adäquate Verständigung nicht gegeben war, z. B.:

„… einige Ärzte, wenn ich hingehe, die sagen du MUSST einen Dolmetscher mitnehmen. Sie sagen, ‚ich untersuche gar nicht‘. Also, das ist auch sein Recht“ (I5: 119).

Beim Zugang zum Gesundheitssystem führten lange Wartezeiten bei der Terminvergabe zu Grenzerfahrungen. Grenzerfahrungen sind deterministisch für negative Versorgungserfahrungen, die die gesundheitliche Situation beeinträchtigen können. So berichtete ein Interviewpartner (I6: 74), dass er trotz des als schwerwiegend wahrgenommenen Gesundheitsproblems lange auf einen Termin warten musste. Ein Befragter (I1: 105) beschrieb, dass er die „Schmerzen erträgt“, bis der Arzttermin „nach langer Zeit“ stattfinden kann, und auch eine weitere Person (I16: 138) gab an, „wochenlang gebraucht“ zu haben, um einen Termin zu bekommen.

14 InterviewpartnerInnen verfügten nicht über ausreichendes Wissen zum Ablauf von Versorgungsprozessen – trotz einer häufigen Inanspruchnahme. Dies zeigte sich z. B. in Hinblick auf Überweisungen, Unterschiede zwischen haus- und fachärztlicher Versorgung oder die beklagten langen Wartezeiten auf Termine. Sie kannten aber in der Regel die für sie relevanten Verfahrensbedingungen. Sie wussten z. B., dass sie eine „AOK-Karte“ brauchen (I13: 276) oder dass es „einen Verein namens BKK gibt“ (I3: 158), um Zugang zum Versorgungssystem zu bekommen. Der Erwerb einer elektronischen Gesundheitskarte (eGK) wurde von 6 Befragten als Erleichterung erlebt, weil damit ein vereinfachter Versorgungszugang ermöglicht wurde. Die mit der Wartezeit auf die eGK verbundenen Zugangsbeschränkungen konnten die Teilhabe am Alltagsleben maßgeblich einschränken. So musste ein Interviewpartner (I11: 270) auf einen Zugang zur Regelversorgung warten, bis er eine Versorgung mit Prothese erhielt:

„Der Ansprechpartner vom Sozialamt hat mir bereits erklärt, dass ich 15 Monate^1^ warten muss. Die Bestellung der Prothesen erfolgte dann mit der AOK-Karte. Es war eine Frage der Zeit.“

Ablehnungserfahrungen wurden von 12 InterviewpartnerInnen gemacht. Weil sich beispielsweise ein Interviewpartner von der Versorgung zurückgewiesen fühlte, nahm er sie nicht mehr in Anspruch, obwohl er ein Bedürfnis nach Schlaftabletten hatte (I9: 83). Die negativen Erfahrungen im Krankheitsfall führten sogar dazu, dass er seine Meinung über die Menschen in Deutschland änderte, da er keinen „humanen Umgang“ erlebt hatte (I9: 169 ff.).

3 Interviewpartnerinnen fühlten sich von der (fach-)ärztlichen Versorgung ausgegrenzt und ungleich behandelt. Die empfundene Dringlichkeit der Behandlung geriet in Konflikt mit den (impliziten) Regeln der Auf- und Annahme in der medizinischen Versorgung. Eine Frau (I14: 270) sagte:

„Zum Beispiel einmal ist meine Tochter krank geworden, ich bin zu ihm [dem Hausarzt] gegangen. Als ich ihm gesagt habe, dass meine Tochter krank ist, sagte er mir ‚warum kommen Sie hier ohne einen Termin vorher abzumachen?‘. Woher soll ich wissen, dass meine Tochter krank sein wird. Der sagte mir ‚ohne Termin werde ich sie nicht untersuchen‘. Ich sagte: ‚Sie sind der Hausarzt‘.“

Die Anhäufung von negativen Erfahrungen kann zu Vertrauensverlusten in die Versorgung führen. So suchten 2 Interviewpartner die Versorgung nicht mehr auf. Ein anderer (I1: 105) fühlte sich dagegen „gezwungen“, die Versorgung in Anspruch zu nehmen, weil seine Schmerzen weiterhin stark waren:

„Einige Ärzte machen die Erkrankung schlimmer und manche helfen tatsächlich. Ich sehe, dass mein Zustand noch nicht behandelt wurde. Ich habe keine andere Wahl, als die Behandlungsmethode beim Arzt zu befolgen. Wenn ich einen Termin mit einem Arzt vereinbare, habe ich kein Gefühl der Genesungssicherheit, sondern einen Zustand der Skepsis und Unsicherheit.“

Die grundlegende Skepsis gegenüber der ärztlichen Versorgung wurde im Narrativ mit den Worten, „manche helfen tatsächlich“, relativiert. In seiner Formulierung zeigte sich eine regelrechte Verwunderung über gelegentliche Behandlungserfolge.

4 InterviewpartnerInnen berichteten von subjektiv wahrgenommenen Fehlbehandlungen als gravierendste Form von Grenzerfahrungen, z. B. (I15: 102):

„Meine Gesundheit hat sich verschlechtert während ich beim ehemaligen Arzt behandelt wurde, eineinhalb Jahre lang. Ich nehme das Medikament täglich, was ein Indikator ist, dass meine Gesundheit schlechter geworden ist.“

Die vermeintlichen Fehlbehandlungen hatten Auswirkungen auf das Alltagsleben von 3 InterviewpartnerInnen, weil sie in ihrer Krankenrolle gefangen blieben und zum Beispiel keine Deutschkurse besucht werden konnten.

### Fremdheit bewältigen

Um Fremdheitserfahrungen zu bewältigen und Zugangsbarrieren abzubauen, wurden sowohl aufseiten der InterviewpartnerInnen als auch aufseiten der VersorgerInnen unterschiedliche Strategien angewendet, um die Versorgung möglichst bedarfs- und bedürfnisgerecht zu gestalten. Die InterviewpartnerInnen bedienten sich unterschiedlicher Strategien zum Abbau von Sprachbarrieren und zur Orientierung im Gesundheitssystem. Bei Professionellen wurden eine empathische Zuwendung und der Aufbau von Versorgungsallianzen als positiv erlebt, die Fremdheitserfahrungen mit und im System verringern können.

#### Strategien der Interviewten

14 InterviewpartnerInnen fanden einen adäquaten Umgang mit den vorhandenen Sprachbarrieren. 10 Personen verfügten bereits über rudimentäre Deutschkenntnisse, die sie in der Gesundheitsversorgung einsetzen konnten. So beschrieb eine Person (I19: 145):

„… Außerdem verstehe ich viel von dem, was gesagt wird. Unter Zwang spreche ich Deutsch, wenn ich sprechen muss.“

Auch ein anderer Interviewpartner (I6: 241) war bemüht, sich mit einem Arzt auf Deutsch zu verständigen. Dieser Verständigungs-„Versuch“ gelang jedoch nur eingeschränkt, sodass seine gesundheitlichen Bedürfnisse unerfüllt blieben. Weibliche Interviewte nutzten zum Teil die Hilfe ihrer Kinder, um sich mit den Professionellen des Gesundheitssystems zu verständigen (z. B. I14: 357). Allerdings kann dies zu Problemen führen, wie eine Interviewpartnerin verdeutlichte, weil ein umfassendes Verständnis ausblieb und sich die Interviewpartnerin nicht angemessen in den Versorgungsprozess einbezogen fühlte (I13: 228). Dolmetschen durch Angehörige kann zur Belastungsprobe werden, wie eine Interviewpartnerin aufzeigte (I4: 466):

„Wenn ich sehr krank bin und starke Schmerzen habe, wollte ich nicht, dass meine Tochter mitkommt, aber ich war gezwungen meine Tochter mitzunehmen … Wenn ich Schmerzen habe, dann würde ich lieber meine Tochter nicht mitnehmen.“

Eine Strategie, die 6 InterviewpartnerInnen anwandten, war das Aufsuchen von ÄrztInnen mit dem gleichen kulturellen und sprachlichen Hintergrund, was jedoch nicht immer zum erhofften Erfolg geführt hat. Während 1 Person den sprachlichen und kulturellen Bezug zu ihrem Hausarzt positiv hervorhob, waren 5 InterviewpartnerInnen sehr unzufrieden mit der Versorgung durch ÄrztInnen aus dem gleichen Sprachraum. Eine Person (I1: 125) schilderte, dass viele Geflüchtete unter dieser Situation „leiden“, aber nicht darüber sprechen:

„Viele Flüchtlinge verlassen arabische Ärzte und wenden sich an deutsche Ärzte.“

Dies bestätigten die Narrationen weiterer InterviewpartnerInnen. Angeführte Gründe waren: fehlendes Vertrauen (z. B. I3: 139, I16: 139), belehrendes Verhalten (z. B. I1: 111) und respektlose Behandlungen (z. B. I14: 269).

Neben den sprachlichen Herausforderungen, die die InterviewpartnerInnen mit den oben beschriebenen Strategien in der Kommunikation zu bewältigen versuchen, müssen sie sich im Versorgungssystem orientieren, um Gesundheitssystemkenntnisse zu gewinnen. So zeigte sich bei einem Befragten (I19), dass er durch die umfassende medizinische und therapeutische Behandlung, die durch einen Dolmetscher begleitet wurde, mit den unterschiedlichen Anlaufstellen im Krankheitsfall vertraut war. Alle anderen InterviewpartnerInnen nutzten eher Freunde, Bekannte, andere Geflüchtete oder das Sozialamt als Informationsquelle, was allerdings als „Glückssache“ bezeichnet wurde, weil den InterviewpartnerInnen unklar blieb, ob korrekte Informationen übermittelt wurden (I1: 143). Insbesondere die befragten Frauen profitierten, sofern sie über Familiennachzug gekommen sind, vom Erfahrungsschatz ihrer Familien. Zudem wurde die Vermittlung in die Gesundheitsversorgung durch die SozialarbeiterInnen in den Unterkünften als hilfreich erlebt (z. B. I2: 467).

#### Strategien der VersorgerInnen

In den Interviews wurden auch Strategien angesprochen, die von den Professionellen im Gesundheitswesen angewandt werden, um sprachliche Barrieren abzubauen. In der stationären Versorgung wurde z. B. auf andere Professionelle wie ÄrztInnen, Pflegepersonal oder medizinische Fachangestellte zurückgegriffen, die über den gleichen sprachlichen Hintergrund wie die InterviewpartnerInnen verfügten. In Hinblick auf die ambulante Versorgung berichtete nur eine Person (I4: 659) davon, dass die kurmandschisprachige Mitarbeiterin einer Gynäkologin beim Einlegen einer Spirale und betreffend die damit verbundenen Kosten gedolmetscht hat. In den anderen Interviews wurde deutlich, dass die Verwendung „einfacher Wörter“ (I3: 153, I17: 201, I19: 196) oder das „Zeit geben, um zu sprechen“ (I5: 94), als hilfreich erlebt wurden, zumindest wenn die InterviewpartnerInnen über Deutschkenntnisse verfügten. Meist konnte damit eine Behandlung initiiert werden, wenngleich es dann bei 4 Personen zum Verdacht auf eine Fehlbehandlung kam.

Eine gute Beziehungsgestaltung zwischen ÄrztInnen und InterviewpartnerInnen wird als hilfreich im Versorgungssystem erlebt, zum Beispiel wenn sich InterviewpartnerInnen als Menschen (und nicht als Flüchtlinge) angenommen fühlen, wie das folgende Zitat nahelegt (I13: 270):

„Der Arzt war geduldig. Er kennt uns nicht und hat uns geholfen. Er geht mit uns nicht als Flüchtlinge um.“

Insbesondere, wenn die empfundene medizinische Unterstützung umfassend war, empfanden die InterviewpartnerInnen die Versorgung als bedürfnisgerecht. So beschrieb eine Person (I14: 294):

„Er [der Hausarzt] macht alles, was er kann.“

3 InterviewpartnerInnen hoben die zeitliche Komponente hervor, 6 InterviewpartnerInnen den empathischen Umgang. Eine Interviewpartnerin erläuterte:

„Immer, wenn ich medizinische Hilfe brauche und zu meinem Arzt gehe, vollzieht der Arzt meine Situation nach. Auch wenn er keine Zeit hätte, versucht er trotzdem eine Lücke zu finden und mich dazwischen zu untersuchen“ (I4: 510).

Ein Interviewter (I2: 791) betonte, dass die ÄrztInnen „geduldig und sorgfältig*“* arbeiteten und seine „Bedürfnisse alle“ befriedigt wurden. Eine gute Arzt/Ärztin-PatientIn-Interaktion schien für ihn die möglichen Sprachbarrieren aufzuwiegen (Zitat Tab. 1; I2: 983).

#### Unterstützung durch das Versorgungssystem

Sofern sich PatientInnen noch im Asylverfahren befanden, konnten sie teilweise auf DolmetscherInnen zurückgreifen, die sie durch das Versorgungssystem begleiteten. Dies wurde von 5 Interviewten als besonders hilfreich erlebt. Einer der Befragten (I2: 305) wertschätzte die umfassende Betreuung und die Erläuterungen durch den Dolmetscher, der ihm vom Sozialamt zur Verfügung gestellt wurde (Zitat Tab. 1; I2: 305). Der Dolmetscher fing in seinem Fall auch Versorgungsherausforderungen auf und kümmerte sich um die gesamten Abläufe. Ein weiterer Interviewter (I1: 151) berichtete von negativen Erfahrungen mit Sprachmittlung und äußerte deshalb die Meinung, dass eine fehlende Qualifizierung, fehlende Sprachkenntnisse, fehlendes medizinisches Wissen und eine fehlende Professionalität Ausschlusskriterien für die Tätigkeit als DolmetscherIn sein sollten, da sie einer guten Behandlung von gesundheitlichen Problemen im Weg stehen.

In 5 Fällen unterstützten die SozialarbeiterInnen der Gemeinschaftsunterkunft bei der Versorgung. Durch ihre Vermittlung von DolmetscherInnen oder Arztterminen wurde die Versorgung als bedürfnisgerecht erlebt. 4 InterviewpartnerInnen berichteten, im Heimatland oder in Transitländern Schwierigkeiten im Zugang zur gesundheitlichen Versorgung gehabt zu haben, was im Vergleich zu einer positiven Bewertung des deutschen Gesundheitssystems führte – trotz der Restriktionen beim Zugang zu Hilfsmitteln, wie ein Interviewpartner beschrieb, oder des Fehlens einer eGK, wie 6 Personen konstatierten.

## Diskussion

Die Gesundheitsversorgung in der Fremde ist mit zahlreichen Herausforderungen für die Befragten verknüpft. Als eine „Besonderheit“ in Deutschland sind hierbei die gesetzlichen Rahmenbedingungen zu nennen, die den Zugang zur Gesundheitsversorgung in den ersten 18 Monaten des Asylverfahrens erschweren (§ 4 AsylbLG). Diese Restriktionen können für die InterviewpartnerInnen die Vertiefung des Gefühls von Fremdheit und eine fehlende gesellschaftliche Teilhabe bedeuten. Gerade durch das Fehlen einer eGK wird der Zugang ins Gesundheitssystem erschwert [15]. Eine Kosten-Nutzen-Analyse des eingeschränkten Versorgungszugangs [36] hat gezeigt, dass – neben den in den Interviews konstatierten individuellen Einschränkungen – Kostensteigerungen durch die gesetzliche Einführung eines restriktiven Versorgungszugangs hervorgerufen wurden. Die Bereitstellung einer eGK während des laufenden Asylverfahrens kann zu einer Verbesserung der Navigation im Versorgungssystem beitragen, weil der Zugang zu FachärztInnen verbessert und die Frequentierung von Notaufnahmen reduziert werden kann [37]. Dies zeigt sich auch in den Aussagen der InterviewpartnerInnen, die einen „schnelleren“ Versorgungszugang mittels eGK konstatieren.

In den Narrativen zeigt sich eine Vielzahl von Grenz- und Ablehnungserfahrungen in der Gesundheitsversorgung. Diese ergeben sich aus: a) einer Diskrepanz zwischen der empfundenen Behandlungsnotwendigkeit und langen Wartezeiten auf einen Termin, b) Sprachbarrieren und fehlender Versorgung mit DolmetscherInnen, c) einer fehlenden Kümmerverantwortung der VersorgerInnen, d) Situationen von Macht- und Hilflosigkeit und e) daraus resultierenden Vertrauensverlusten in die Versorgung. Diskriminierungserfahrungen und eine schlechte Beziehung zwischen den Professionellen im Versorgungssystem und den PatientInnen mit Fluchterfahrungen werden in der Literatur ebenfalls problematisiert [17, 18, 38, 39]. Wie Jensen et al. [39] zeigen, hängt die Zufriedenheit mit der Behandlung maßgeblich mit der TherapeutInnen-PatientInnen-Beziehung zusammen. Wichtig ist dabei eine empathische Grundhaltung der Versorgenden, die nicht in allen Fällen und zu jeder Zeit von den InterviewpartnerInnen empfunden wurde. Dies war unabhängig vom kulturellen Hintergrund der behandelnden ÄrztInnen. Einige InterviewpartnerInnen berichteten von fehlenden Vertrauensverhältnissen und enttäuschten Erwartungen bei der Behandlung durch ÄrztInnen, die über den gleichen kulturellen und sprachlichen Hintergrund verfügen. 2 (quantitative) Studien aus den USA konnten dagegen zeigen, dass eine ethnische Konkordanz zwischen ÄrztInnen und PatientInnen zu besseren gesundheitlichen Outcomes führen kann [40, 41].

Sprachbarrieren spielen eine große Rolle in der Gesundheitsversorgung der befragten Geflüchteten. Hier manifestiert sich die Fremdheit im Versorgungssystem am deutlichsten. Diese Erkenntnis schließt an eine Vielzahl anderer Studien an (z. B. [13, 42, 43]). Sprachbarrieren führen dazu, dass gesundheitliche Bedürfnisse nicht ausreichend befriedigt werden. Teilweise bedingen sie Grenzerfahrungen, weil die PatientInnen sich nicht äußern können und somit nicht an Versorgungsentscheidungen beteiligt werden (können). Dadurch wird die Selbst- und Mitbestimmung eingeschränkt. Die meisten InterviewpartnerInnen fanden einen für sie adäquaten Umgang mit den sprachlichen Herausforderungen. Denn Menschen mit Fluchtgeschichte bekommen oft keine/n DolmetscherIn zur Verfügung gestellt [6]. Sofern ein/e DolmetscherIn vorhanden ist, ist er/sie manchmal nur unzureichend für seine Aufgabe qualifiziert. In den Interviews wird thematisiert, dass insbesondere Kinder als DolmetscherInnen einbezogen werden, was für diese und für die PatientInnen mit emotionalen Belastungen verbunden sein [44]. Seitens der PatientInnen kann dies zudem zu Loyalitätskonflikten gegenüber den Angehörigen führen [45]. Derzeit zeigen sich also zahlreiche Defizite im Einsatz von DolmetscherInnen in der Versorgung. Es fehlen Qualitätssicherungssysteme; zudem sind die Ausbildung von DolmetscherInnen und die Anforderungen an sie uneinheitlich geregelt. Überdies mangelt es derzeit an einer rechtlichen Grundlage für die Finanzierung von Dolmetscherleistungen in der Regelversorgung der gesetzlichen Krankenversicherung (GKV), wodurch es zur Ungleichbehandlung von PatientInnen mit unzureichenden Deutschkenntnissen kommen kann [21].

Robertshaw et al. [46] erörtern in einem systematischen Review die Chancen und Herausforderungen für Professionelle des Gesundheitssystems bei der Versorgung von Geflüchteten. Eine wichtige Rolle spielen die Beziehungsebene zwischen Arzt/Ärztin und PatientIn, das Vertrauen zueinander, die Kommunikation, das kulturelle Verstehen, die gesundheitlichen Belange der Asylsuchenden und zeitliche Kapazitäten. Für eine gute Versorgung ist es zudem zentral, die Lebenssituation von Menschen mit Fluchtgeschichte zu berücksichtigen und gesundheitsförderliche Bedingungen zu identifizieren. Dies legen auch die Aussagen der InterviewpartnerInnen aus NutzerInnen-Perspektive nahe. Die Beziehungsebene zwischen VersorgerIn und PatientIn erwies sich in den Interviews als besonders relevant für die Erfüllung der Versorgungsbedürfnisse. In den Aussagen der InterviewpartnerInnen zeigte sich, dass empfundene Empathie durch die Professionellen des Gesundheitssystems, ein gutes Vertrauensverhältnis zwischen Arzt/Ärztin und PatientIn, die Erfüllung vorhandener gesundheitlicher Bedürfnisse und ein schnelles Handeln im Krankheitsfall als besonders hilfreich wahrgenommen wurden. Entscheidend ist deshalb, die Gesundheitsversorgung nicht nur aus einer defizitorientierten Perspektive zu betrachten, sondern gezielt (vorhandene) systemische Ressourcen in den Blick zu nehmen. Tab. 2 fasst die identifizierten Versorgungsdefizite zusammen und erläutert mögliche Gegenmaßnahmen, um die Herausforderungen für Geflüchteten im Zugang zur Gesundheitsversorgung abzumildern.

**Table 2:** 

Identifizierte Versorgungsdefizite	Gegenmaßnahmen
Sprachbarrieren, die die Selbst- und Mitbestimmung einschränken	Flächendeckende Einführung von (medizinisch) qualifizierten DolmetscherInnen
Fehlendes Wissen über Versorgungsprozesse und Unterschiede über haus- und fachärztliche Versorgung	Stärkung von Projekten zur Ausbildung von GesundheitsmediatorInnen, damit PatientInnen zukünftig besser über die Gesundheitsversorgung informiert werden können
Restriktiver Versorgungszugang, der die Teilhabe am Alltagsleben einschränken kann	Abbau von strukturellen Zugangsbarrieren durch Abschaffung des restriktiven Versorgungszugangs
Ablehnungserfahrungen	Stärkung der Beziehung zwischen ÄrztInnen und PatientInnen durch eine Sensibilisierung für die gesundheitlichen Bedarfe und Bedürfnisse geflüchteter PatientInnen
	Stärkere Ressourcenorientierung in der Behandlung und Versorgung von Menschen mit Fluchtgeschichte

### Limitationen

Die Analyse der Interviews geschah im Wesentlichen durch die Erstautorin, wodurch es zu einer Einschränkung bei der Dateninterpretation gekommen sein könnte. Eine kommunikative Validierung der Interpretationen mit den InterviewpartnerInnen – ein Verfahren, um die Validität der qualitativen Daten zu erhöhen [47] –, konnte aufgrund fehlender finanzieller und sprachlicher Ressourcen nicht stattfinden. Die Erhebungs- und Auswertungsmethode erwies sich als angemessen, da es sich um dolmetschergestützte Interviews handelte, diese also in trialogischen und damit im Vermittlungsakt „unterbrochenen“ Gesprächen stattgefunden haben. Der Einsatz von SprachmittlerInnen könnte dazu geführt haben, dass bestimmte Fragen und Aussagen nicht adäquat übersetzt wurden und damit wichtige Informationen verloren gegangen sind. Auch die Gesprächssteuerung war an manchen Stellen nur schwer möglich, weil vertiefende Nachfragen nicht realisierbar waren. Zudem müssen mögliche Einflüsse der Anwesenheit und des Geschlechts der DolmetscherInnen auf den Interviewverlauf kritisch angemerkt werden (vgl. [34]).

## Fazit

Geflüchtete Menschen erleben in Deutschland eine Vielzahl von Herausforderungen im Zugang zur Gesundheitsversorgung. Gleichzeitig können sie auf unterschiedliche individuelle, soziale und strukturelle Ressourcen zurückgreifen, um Zugangsbarrieren abzubauen. Weiterführende Forschung ist notwendig, um gezielt(er) Ressourcen von Menschen mit Fluchtgeschichte bei der Inanspruchnahme der Gesundheitsversorgung zu berücksichtigen. Dabei sollten auch weitere Flüchtlingsgruppen (z. B. ukrainische Geflüchtete oder Geflüchtete ohne dauerhafte Bleibeperspektive) einbezogen werden.

## Footnotes

1 Zum Zeitpunkt der Interviews hatten InterviewpartnerInnen im Asylverfahren oder mit unklarer Bleibeperspektive bereits nach 15 Monaten Zugang zu Analogleistungen. Mit dem sog. Geordnete-Rückkehrer-Gesetz wurde § 2 Abs. 1 verändert und die Leistungsberechtigung zum 01.09.2019 von 15 auf 18 Monate erhöht.
